# Surgical interventions for symptomatic knee osteoarthritis: a network meta-analysis of randomized control trials

**DOI:** 10.1186/s12891-023-06403-z

**Published:** 2023-04-22

**Authors:** Geng Bin, Liu Jinmin, Tian Cong, Tang Yuchen, Zhang Xiaohui, Xia Yayi

**Affiliations:** 1 Department of Orthopaedics, Lanzhou University Second Hospital, Lanzhou, Gansu China; 2 Gansu Province Clinical Research Center for Orthopaedics, Lanzhou, Gansu China; 3 Lanzhou University Second Hospital, #82 Cuiyingmen, Lanzhou, Gansu, 730000 China

**Keywords:** Total knee arthroplasty, Unicompartmental knee arthroplasty, High tibial osteotomy, Bicompartmental knee arthroplasty, Bi-unicompartmental knee arthroplasty, Knee joint distraction

## Abstract

**Background:**

Multiple surgical interventions exist for the treatment of symptomatic knee osteoarthritis, but the surgeon and patient may often have difficulty deciding which interventions are the best option.

**Methods:**

We conducted a systematic review to identify randomized clinical trials (RCTs) that compared complications, revisions, reoperations, and functional outcomes among TKA (total knee arthroplasty), UKA (unicompartmental knee arthroplasty), HTO (high tibial osteotomy), BCA (bicompartmental knee arthroplasty), BIU (bi-unicompartmental knee arthroplasty), and KJD (knee joint distraction). The PubMed, Embase, and Cochrane databases were reviewed for all studies comparing two or more surgical interventions. Direct-comparison meta-analysis and network meta-analysis (NMA) were performed to combine direct and indirect evidence. The risk of bias was assessed using the revised Cochrane risk of bias tool for RCTs.

**Results:**

This NMA and systematic review included 21 studies (17 RCTs), with a total of 1749 patients. The overall risk-of-bias assessment of the RCTs revealed that 7 studies had low risk, 5 had some concerns, and 9 had high risk. SUCRA (the surface under the cumulative ranking curve) rankings revealed that KJD had the greatest risk of appearing postoperative complications, revisions, and reoperations, and UKA or TKA had the lowest risk. The majority of comparisons among various treatments showed no difference for functional outcomes.

**Conclusion:**

Each surgical intervention is noninferior to other treatments in functional outcomes, but UKA and TKA are better options to treat OA according to SUCRA rankings by comparing complications, revisions, and reoperations. KJD is an imperfect option for treating OA. Other treatments should be carefully considered for each patient in accordance with their actual conditions. However, this conclusion is limited by the selection of reviewed publications and individual variation of surgical indications for patients.

**Trial registration:**

This study was registered with Research Registry (reviewregistry1395).

**Supplementary Information:**

The online version contains supplementary material available at 10.1186/s12891-023-06403-z.

## Introduction

Knee osteoarthritis (OA) is a common joint disease in older patients that may cause severe pain and lead to an increasing financial burden and a reduced quality of life [1]. Although knee OA may involve any one or all 3 compartments, up to 30% of patients have evidence of only single compartmental degeneration [2].

The optimal surgical treatment for OA of the knee remains in question. To date, several surgical solutions have been proposed to address OA, such as TKA (total knee arthroplasty), UKA (unicompartmental knee arthroplasty), HTO (high tibial osteotomy), BCA (bicompartmental knee arthroplasty), BIU (bi-unicompartmental knee arthroplasty), or KJD (knee joint distraction), disagreements often exist between surgeons regarding the best choice of procedure and the best decision for patients with OA [3].

For example, although TKA is a primary surgical treatment for OA, as many as 15-20% of patients are dissatisfied with their surgical outcome [4–6]. Good or excellent long-term results with high patient satisfaction were reported for UKA, but its survival has been found to be inferior to that of TKA [7–10]. Unlike TKA and UKA, HTO accomplishes the reconstruction of joint function by correcting varus malalignment; however, some HTOs may need a conversion to TKA due to the progression of OA, and TKA following HTO has worse outcomes and higher complications [11–13]. Compared to TKA, BCA is a less invasive procedure but might have a relevant change in the leg axis and poor long-term survivorship [14, 15]. BIU demonstrates good functional outcomes, but data on its long-term outcomes remain limited [16]. KJD is a new surgical joint-preserving treatment that also appears to be associated with joint tissue regeneration, but relevant evidence is sparse [17]. Hence, for surgical interventions for knee OA, there is high variation in treatment choice and little robust evidence to guide selection.

The purpose of the present study was to perform a systematic review and network meta-analysis (NMA) of randomized control trials (RCTs) comparing different surgical treatments (TKA, UKA, HTO, BCA, BIU, and KJD) and assessing their complications, revisions, reoperations, and functional outcomes.

**Table 1:** Study Characteristics

Study	Design	Country	Published journals	Interventions	Recruited Recruited Patients (n)	Randomised Patients (n)	Analysed Patients (Knees), n	Age (mean)	Sex (F/M)	Flollw-up
1. Wu 2022	RCTs	China	Orthop Surg	TKA	220	60	60 (60)	63	NA	3 years
				UKA		120	119 (119)	64	NA	
2. Knifsund 2021	RCTs	Finland	BMJ Open	TKA	143	71	70 (70)	62.9	30/41	2 years
				UKA		72	69 (69)	63.3	33/39	
3. Beard 2019	RCTs	UK	Lancet	TKA	962	264	269 (269)	64.7	222/306	5 years
				UKA		264	245 (245)	65.2		
4. Kulshrestha 2017	RCTs	India	J Arthroplasty	TKA	100	40	36 (72)	62.19	56/16	2 years
				UKA		40	36 (72)	59.72		
5. Murray 2014	RCTs	UK	Health Technology Assessment	TKA	34	17	16 (16)	67	19/15	10 years
				UKA		17	18 (18)	66		
6. Sun 2012	RCTs	China	Knee	TKA	62	28	28 (56)	61	37/19	4.3 years
				UKA		28	28 (56)	60		
7. Costa 2011	RCTs	USA	J Knee Surg	TKA	34	17	17 (34)	73	15/19	5 years
				UKA		17	17 (34)			
8. Newman 2009*	RCTs	UK	J Bone Joint Surg Br	TKA	94	47	26 (27)	69.8	55/39	15 years
				UKA		47	24 (28)	69.6		
9. Weale 1999*	RCTs	UK	J Bone Joint Surg Br	TKA	100	47	40 (45)	69.8	56/38	5 years
				UKA		45	38 (43)	69.6		
10. Newman 1998*	RCTs	UK	J Bone Joint Surg Br	TKA	100	47	40 (46)	69.8	56/38	5 years
				UKA		45	40 (45)	69.6		
11. Börjesson 2005	RCTs	Sweden	Knee	UKA	100	50	22 (22)	63	19/21	5 years
				HTO		50	18 (18)	63		
12. Stukenborg-Colsman 2001	RCTs	Germany	Knee	UKA	60	28	28 (30)	67	35/25	7–10 years
				HTO		32	32 (32)	67		
13. weidenhielm 1992	RCTs	Sweden	Clin Biomech	UKA	53	28	28 (28)	63	28/25	1 year
				HTO		25	25 (25)	63		
14. Goh 2020*	RCTs	Singapore	Knee	TKA	121	22	17 (17)	63.1	37/11	10 years
				BCA		26	22 (22)	63.8		
15. Yeo 2015*	RCTs	Singapore	Knee	TKA	121	22	20 (20)	63.1	37/11	5years
				BCA		26	22 (22)	63.8		
16. Schrednitzki 2020	RCTs	Germany	J Arthroplasty	TKA	1289	40	38 (38)	63.55	59/21	5 years
				BCA		40	37 (37)	65.25		
17. Engh 2014	RCTs	USA	J Arthroplasty	TKA	50	25	25 (25)	58.3	NA	2 years
				BCA		25	25 (25)	60.3		
18. Blyth 2021	RCTs	UK	Bone Joint J	TKA	209	38	39(39)	70.4	38/38	1 year
				BIU		42	32 (32)	68.7		
19. Jansen 2021*	RCTs	Netherlands	Cartilage	TKA	129	40	34 (34)	55.4	55/59	2 years
				HTO		46	41 (41)	49.3		
				KJD		43	39 (39)	51.2–55.7		
20. van der Woude 2017 A*	RCTs	Netherlands	Knee Surg Sports Traumatol Arthrosc	HTO	69	46	45 (45)	49.4	24/43	1 year
				KJD		23	22 (22)	51.2		
21. van der Woude 2017 B*	RCTs	Netherlands	Bone Joint J	TKA	60	40	36 (36)	55.2		
				KJD		20	20 (20)	54.9	34/22	1 year

RCTs: Randomized Control Trials, TKA: Total Knee Arthroplasty, UKA: Unicompartmental Knee Arthroplasty, HTO: High Tibial Osteotomy, BCA: Bicompartmental Knee Arthroplasty, BIU: Bi-unicompartmental Knee Arthroplasty, KJD: Knee Joint Distraction, NA: Not Applicable, F/M: Female/Male. *: Same RCT.

## Materials and methods

### Study selection

This NMA is reported following the standards proposed by PRISMA (Preferred Reporting Items for Systematic Reviews and Meta-Analyses) guidelines [18]. This study was registered with Research Registry (reviewregistry1395). Two researchers independently performed the literature search, and any discrepancies were reappraised and arbitrated by a third investigator. The titles and abstracts were screened, and the full-text was reviewed whenever necessary to evaluate the eligibility of each study. The search was performed in PubMed, Embase, and the Cochrane Library Database through March 20, 2022. The search terms included: total knee replacement, unicompartmental joint replacement, high tibial osteotomy, bicompartmental knee arthroplasty, bi-unicompartmental knee arthroplasty, and knee joint distraction. Furthermore, the corresponding references of all included studies were manually screened according to the selection criteria for consideration of inclusion in the study. There was no restriction regarding the publication date.

### Eligibility criteria

We included all multiarm RCTs of human subjects that compared complications, revisions, reoperations, and functional outcomes among TKA, UKA, HTO, BCA, BIU, or KJD with a minimum 1-year follow-up.

The exclusion criteria were as follows: (1) nonrandomized and nonclinical studies, (2) single-arm clinical trials, (3) case reports and series, (4) conference abstracts, and (5) non-English language publications.

### Data extraction and risk of bias assessment

All relevant information regarding the study characteristics was collected: study design, population characteristics, surgical approaches, indications, prosthesis, clinical outcomes, and follow-up time points. Two independent reviewers extracted data using a predetermined data sheet and evaluated the risk of bias using the revised Cochrane risk of bias tool for randomized trials (ROB 2) [19]. In cases of disagreement, a third reviewer was consulted to reach an agreement. The clinical outcomes evaluated included complications, revisions, reoperations, and functional outcomes. The complications were defined as any issues related to the surgical procedure that might require readmission, reoperation, additional treatment or longer hospital stays. Postoperative death from any relevant cause was also considered as a complication. Revisions of UKA, HTO, BCA, BIU and KJD were defined as failures equating to revision to TKA. The reoperations were defined as any unscheduled operations resulting from surgical site complications, including irrigation and wound revision, debridement, implant revision, open reduction internal fixation, and others.

**Fig. 1:**
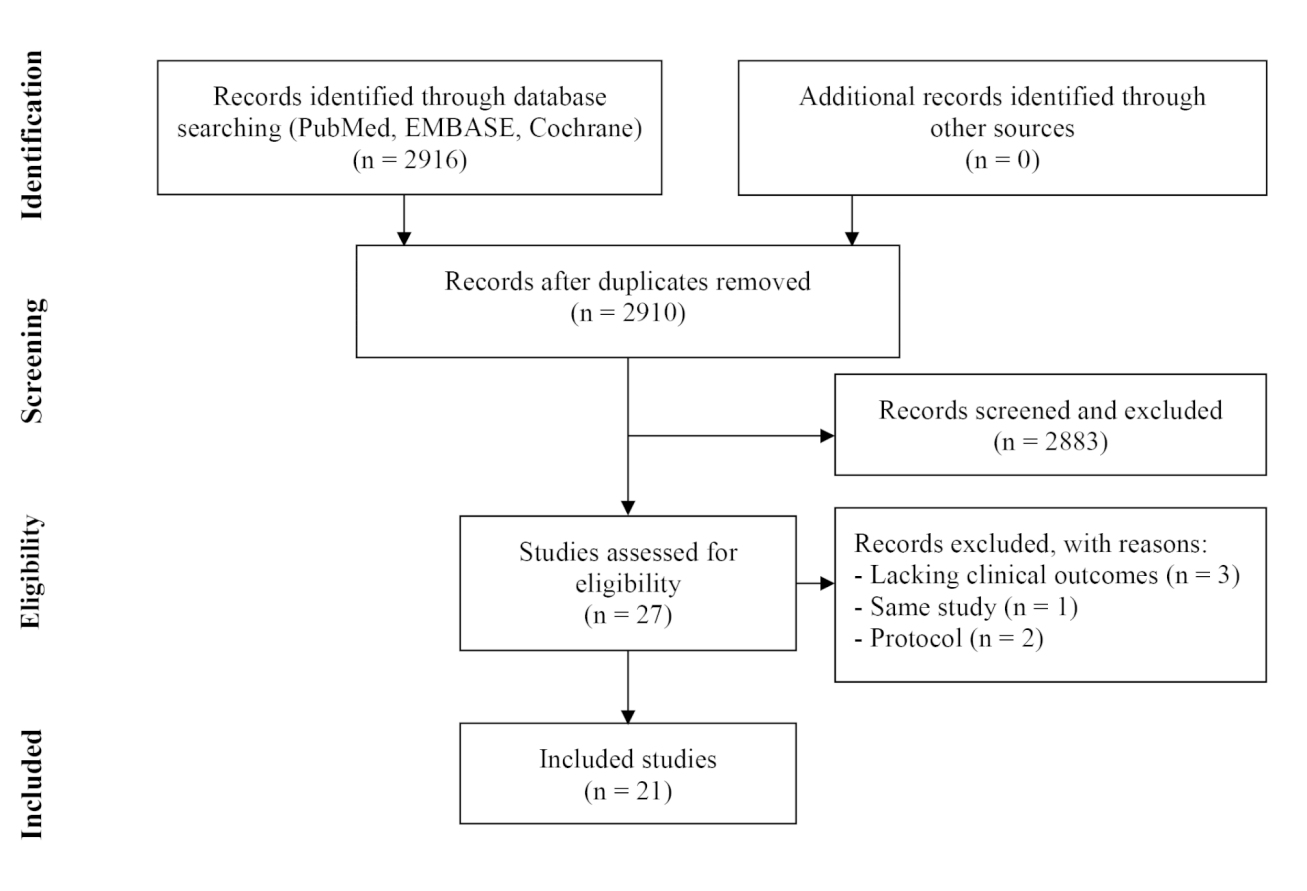
PRISMA flow diagram

**Fig. 2:**
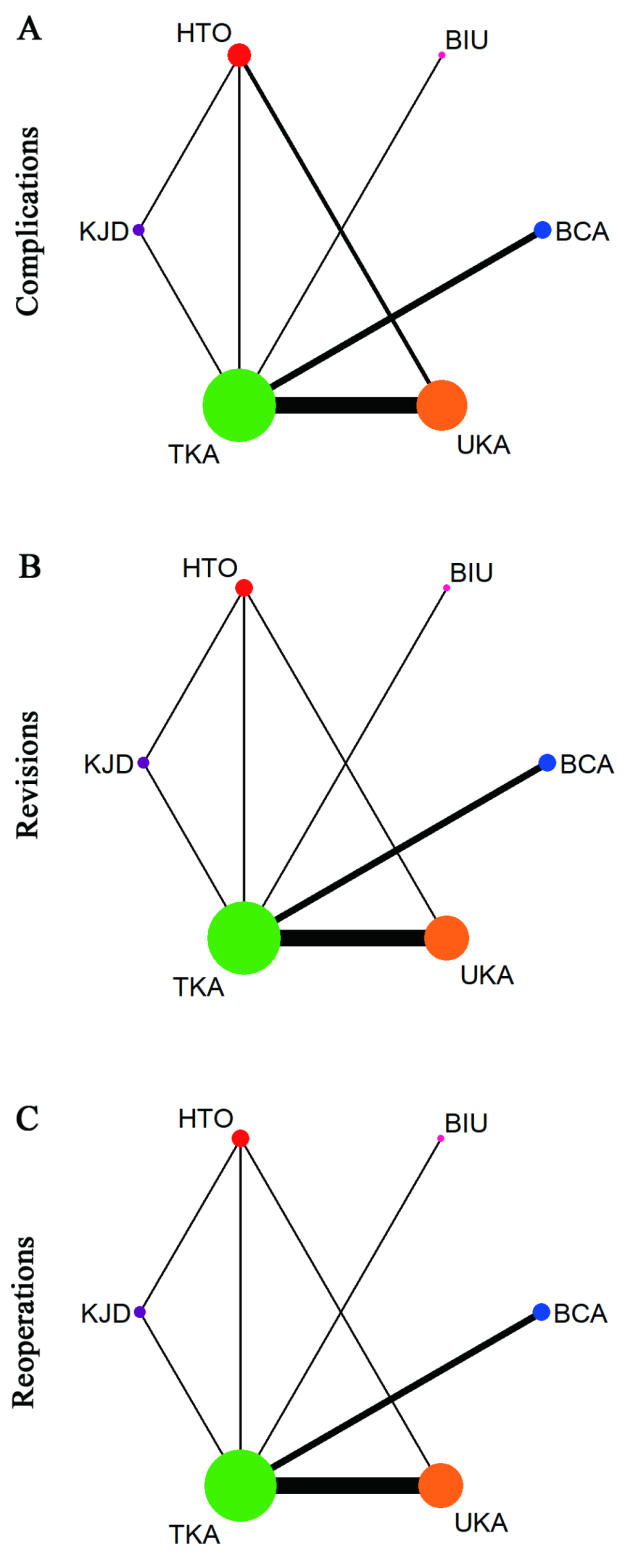
**a-c** Network geometry of different surgical interventions for comparisons of complications (**a**), revisions (**b**), and reoperations (**c**)

**Table 2:** Function Outcomes

Study, Year	Interventions	Prosthesis	Function Outcomes	Results
1. Wu 2022	TKA	Sigma PFC cemented TKA (DePuy, Warsaw, IN, USA)	HSS, WOMAC, KSS, VAS, OKS, ROM, FJS	3 years: **ND** (VAS and KSS function); **SD** (HSS, ROM WOMAC, OKS, FJS)
	UKA	(1) Oxford phase 3 (Zimmer Biomet, Warsaw, IN, USA); (2) Link medial FB UKA (Link, Endo-model Sled, Germany).		
2. Knifsund 2021	TKA	Triathlon (Stryker, Mahwah, New Jersey, USA) cruciate-retaining device.	OKS, KOOS, 15D, KSS, ROM	2 years: **ND** (OKS, KOOS pain, KOOS function, KOOS quality of life and 15D); **SD** (KOOS symptoms, ROM)
	UKA	Oxford phase 3 mobile-bearing device.		
3. Beard 2019	TKA	(1) Low Contact Stress or PFC/Sigma (DePuy Orthopaedics Inc.); (2) Vanguard or NexGen® (Zimmer Biomet); (3) Triathlon® Knee System (Stryker, Mahwah, NJ, USA); (4) Genesis or Genesis (Smith & Nephew, Memphis, TN, USA); (5) ACS® (Implantcast, Buxtehude, Germany); (6) EUROS (Euros SAS, La Ciotat, France); (7) AllPoly (Zimmer Biomet); (8) Oxinium (Smith & Nephew)	OKS, KSS, UCLA, HAAS, EQ-5D-3 L, EQ-5D-VAS	5 years: **ND** (OKS, EQ-5D-3 L, HAAS, UCLA, KSS); **SD** (EQ-5D-VAS)
	UKA	1. Oxford® Partial Knee (Zimmer Biomet, Warsaw, IN, USA) ; 2. Zimmer or Vanguard® (Zimmer Biomet); 3. M/G® Unicompartmental Knee System (Zimmer Biomet); 4. Uniglide™ (Corin Group, Cirencester, UK); 5. AMC (Corin Group); 6. DePuy (DePuy Orthopaedics Inc., Warsaw, IN, USA); 7. Mathys (Mathys Ltd, Bettlach, Switzerland); 8. Medacta (Medacta International, Castel San Pietro, Switzerland); 9. Sigma (DePuy Orthopaedics Inc.).		
4. Kulshrestha 2017	TKA	A cemented, posterior-stabilized implant (None Details)	KOS-ADLS, HAAS, OKS, EQ-5D-VAS	2 years: **ND**
	UKA	A fixed bearing design (None Details)		
5. Murray 2014	TKA	**NA**	OKS, EQ-5D, SF-12 PCS, SF-12 MCS	10 years: **ND**
	UKA	**NA**		
6. Sun 2012	TKA	AGC, Biomet	KSS, ROM, VAS	4.3 years: **ND**
	UKA	Oxford Biomet, Warsaw, IN, USA		
7. Costa 2011	TKA	Scorpio1, cruciate retaining system (Stryker Orthopaedics, Mahwah, NJ)	KSS-functional or clinical	5 years: **ND**
	UKA	(1) EIUS1 fixed bearing system (Stryker Orthopaedics, Mahwah, NJ); (2) Zimmer1 Unicompartmental High-Flex Knee System (Zimmer Inc., Warsaw, IN).		
8. Newman 2009	TKA	Posterior-cruciate-preserving Kinematic Modular TKR (Howmedica, Rutherford, New Jersey)	BKS, ROM	15 years: **ND**
	UKA	St Georg Sled UKR (Waldemar Link, Hamburg, Germany)		
9. Weale 1999	TKA	Posterior-cruciate-preserving Kinematic Modular TKR (Howmedica, Rutherford, New Jersey)		5 years: **ND** (BKS), **SD** (ROM)
	UKA	St Georg Sled UKR (Waldemar Link, Hamburg, Germany)		
10. Newman 1998	TKA	Posterior-cruciate-preserving Kinematic Modular TKR (Howmedica, Rutherford, New Jersey)		
	UKA	St Georg Sled UKR (Waldemar Link, Hamburg, Germany)		
11. Börjesson 2005	UKA	Brigham prosthesis	BOA, Borg CR-10, ROM	5 years: **ND**
	HTO	A Coventry closing wedge osteotomy		
12. Stukenborg-Colsman 2001	UKA	Tübingen pattern, Aesculap®	KSS-functional or clinical, ROM	7–10 years: **ND**
	HTO	A modified osteotomy of Coventry and Weber		
13. weidenhielm 1992	UKA	Brigham prosthesis	BOA	1 year: **ND**
	HTO	A closing wedge osteotomy of Coverntry		
14. Goh 2020	TKA	DePuy Sigma®, Fixed Bearing Knee System, Warsaw, Indiana, United States	OKS, AKSS, SF-36 (PCS, MCS)	10 years: **ND**
	BCA	DePuy Preservation™ Unicompartmental Knee, Warsaw, Indiana, United States and DePuy Sigma® High Performance Partial Knee, Warsaw, Indiana, United States		
15. Yeo 2015	TKA	DePuy Sigma®, Fixed Bearing Knee System, Warsaw, Indiana, United States	OKS, AKSS, SF-36 (PCS, MCS)	5 years: **ND**
	BCA	DePuy Preservation™ Unicompartmental Knee, Warsaw, Indiana, United States and DePuy Sigma® High Performance Partial Knee, Warsaw, Indiana, United States		
16. Schrednitzki 2020	TKA	Innex System (Zimmer Biomet, Warsaw, IN).	KSS-functional or clinical, OKS, UCLA, FJS, TUGT, ROM	5 years: **ND** (KSS, OKS, UCLA, FJS, TUGT); **SD** (ROM)
	BCA	Sigma HP Partial Knee System (DePuy, Warsaw, IN)		
17. Engh 2014	TKA	Genesis II TKA components (Smith & Nephew, Andover, MA)	KSS, OKS, FAT	2 years: **ND**
	BCA	Journey Deuce (Smith & Nephew, Andover, MA)		
18. Blyth 2021	TKA	NexGen LPS implant (Zimmer, USA)	VAS, OKS, KSS, FJS, EQ-5D-3 L, UCLA, HADS, ROM, TUGT, SCT	1 year: **ND**
	BIU	Medial and lateral Restoris MCK (MultiCompartmental Knee)		
19. Jansen 2021	TKA	Genesis II posterior stabilised system (Smith & Nephew, Warsaw, Indiana)	WOMAC, KOOS, VAS, EQ-5D-3 L, SF-36 (PCS and MCS)	2 years: **KJD VS TKA ND** (WOMAC), **SD** (KOSS, VAS, EQ-5D, SF-36); **KJD VS HTO ND** (WOMAC, KOSS, VAS, EQ-5D, SF-36)
	HTO	Opening-wedge osteotomy (TomoFix medial high tibial plates and screws (DePuy Synthes, Switzerland) or Synthes locking compression plate (LCP) system (DePuy Synthes, Switzerland))		
	KJD	Triax proof-of-concept external distraction device (Stryker, Kalamazoo, Michigan)		
20. van der Woude 2017 A	HTO	Opening-wedge osteotomy (TomoFix medial high tibial plates and screws (DePuy Synthes, Switzerland) or Synthes locking compression plate (LCP) system (DePuy Synthes, Switzerland))	WOMAC, KOOS, VAS, EQ-5D-3 L, SF-36 (PCS and MCS)	1 year: **ND** (WOMAC, KOSS, VAS, EQ-5D, SF-36 MCS), **SD** (VAS, SF-36 PCS)
	KJD	Triax proof-of-concept external distraction device (Stryker, Kalamazoo, Michigan)		
21. van der Woude 2017 B	TKA	Genesis II posterior stabilised system (Smith & Nephew, Warsaw, Indiana)	WOMAC, KOOS, VAS, EQ-5D-3 L, SF-36 (PCS and MCS)	1 year: **ND** (WOMAC, KOSS, VAS, EQ-5D, SF-36), **SD (ROM)**
	KJD	Triax proof-of-concept external distraction device (Stryker, Kalamazoo, Michigan)		

TKA: Total Knee Arthroplasty, UKA: Unicompartmental Knee Arthroplasty, HTO: High Tibial Osteotomy, BCA: Bicompartmental Knee Arthroplasty, BIU: Bi-unicompartmental Knee Arthroplasty, KJD: Knee Joint Distraction, HSS: Hospital for Special Surgery Knee Score, WOMAC: The Western Ontario and McMaster Universities Index, KSS: Knee Society Score, VAS: Visual Analog Scale, OKS: Oxford Knee Score, ROM: Range of motion, FJS: The Forgotten Joint Score, KOOS: Knee injury and Osteoarthritis Score, 15D: 15-Dmensional Instrument, EQ-5D-3 L: EuroQol EQ-5D-3 L, UCLA: University of California, Los Angeles Activity score, HAAS: High Activity Arthroplasty Score, KOS-ADLS: Knee Outcome Survey-Activities of Daily Living Scale, SF-12: Short Form Questionnaire-12 Items, BKS: Bristol Knee Score, BOA: British Orthopaedic Association Score, SF-36: Short Form 36, TUGT: Timed-Up-and-Go Test. FAT: Functional Assessment Test, HADS: Hospital Anxiety and Depression Scale, SCT: stair climbing test, NA: Not Applicable, ND: No Significant Difference, SD: Significant Difference

**Fig. 3:**
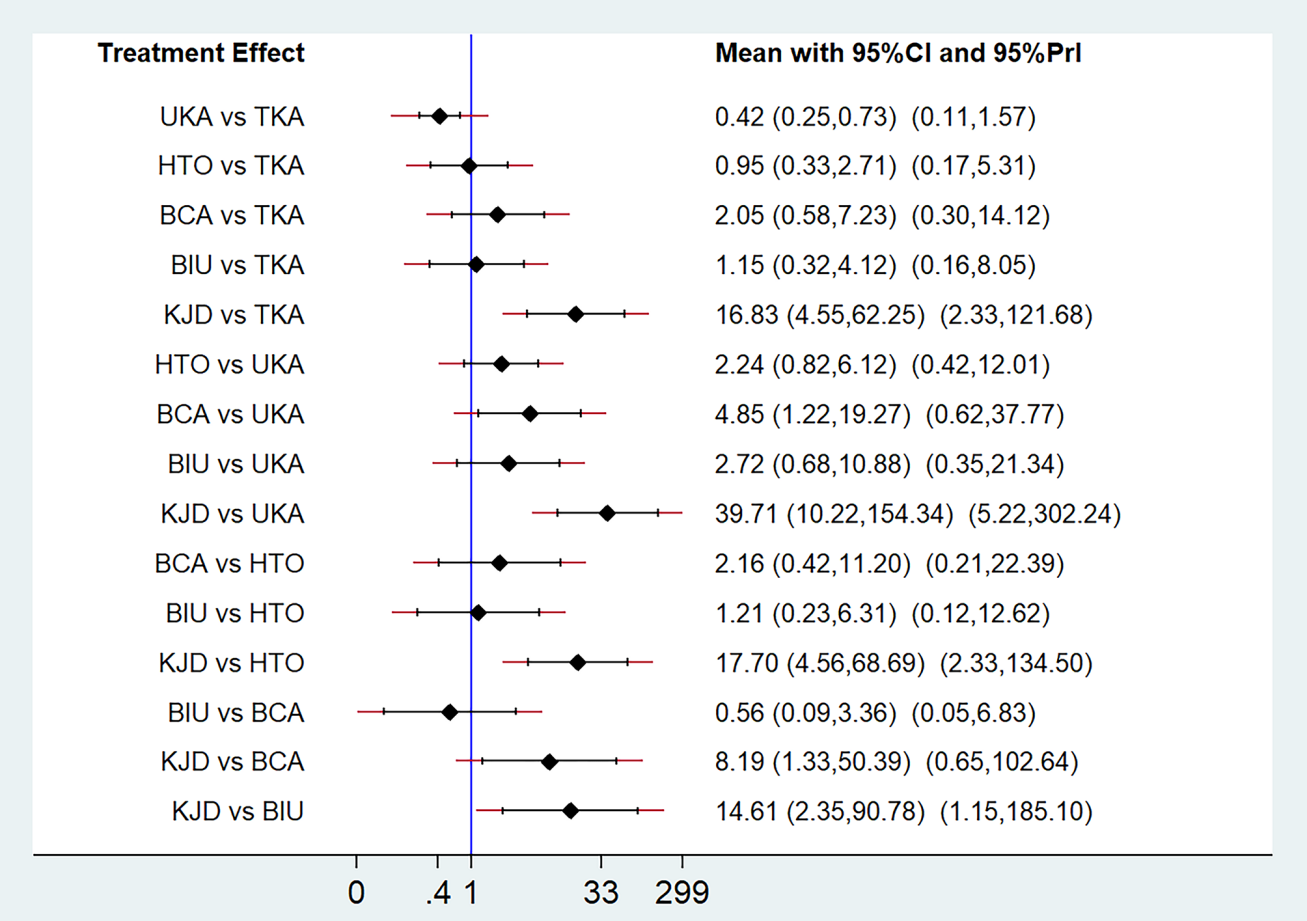
NMA results for complications. The treatments are compared in a forest plot. The vertical reference line centered at 1 indicates statistical equivalence

**Fig. 4:**
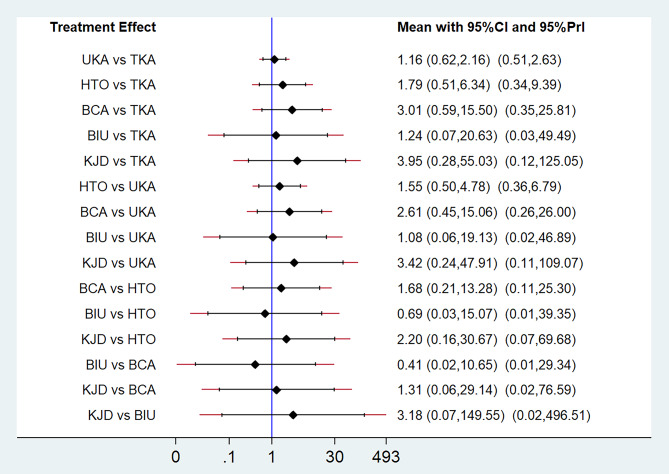
NMA results for revisions. The treatments are compared in a forest plot. The vertical reference line centered at 1 indicates statistical equivalence

### Statistical analysis

For dichotomous outcomes, the relative effect sizes were reported as odds ratios (ORs) with 95% confidence intervals (CIs). When the 95% CI of the OR contained 1, the comparison was considered to have no statistical significance. For direct comparisons, a conventional meta-analysis was conducted to synthesize the results using random-effects models as sensitivity analyses (Supplementary Figs. 1–3). An NMA using a frequentist approach with a random effects model was used to estimate direct and indirect comparisons. NMA aims to test whether superiority exists for one of the comparator interventions. The potential inconsistency between the indirect and direct comparisons was inferred by global inconsistency, local inconsistency (a node-splitting approach), and loop inconsistency. Heterogeneity was quantified using the tau value, and *P* < 0.05 was considered statistically significant. A global network diagram was used for each prespecified outcome to demonstrate direct comparisons between interventions. A comparative hierarchy was obtained by calculating the relative ranking probabilities between the effects of all interventions for the target outcomes and SUCRA (the surface under the cumulative ranking curve). The SUCRA value showed the percentage of procedural efficacy and safety of each treatment and ranged from 0 to 100%. Hence, the larger the SUCRA value, the higher the rank of the intervention, indicating generally a better or worse effect. The contribution plot for the network is summarized in Supplementary Fig. 4, and the size of each circle is proportional to the weight attached to each direct summary effect for the estimation of each network summary effect. The comparison-adjusted funnel plot was used to assess the possibility of publication bias (Supplementary Fig. 5). All statistical analyses were performed using Stata 14 software (StataCorp LP, USA).

## Results

### Literature review and risk of bias assessment

The initial literature search identified a total of 2916 studies. Then, 2883 studies were excluded after screening the titles and abstracts. Twenty-one studies were included in this review [20–40]. Due to different durations of follow-up or clinical outcomes, studies 8–10 (one RCT) [27–29], 14–15 (one RCT) [33, 34] and 19–21 (two RCTs) [38–40] were all included to avoid omitting any clinical outcome. Finally, 21 studies were included in this review, with a total of 1749 patients. There were 709 patients treated using TKA, 711 treated using UKA, 153 treated using HTO, 91 treated using BCA, 42 treated using BIU, and 43 treated using KJD. The details of the literature review are presented in Fig. 1. The characteristics of the included studies are summarized in Table 1.

**Fig. 5:**
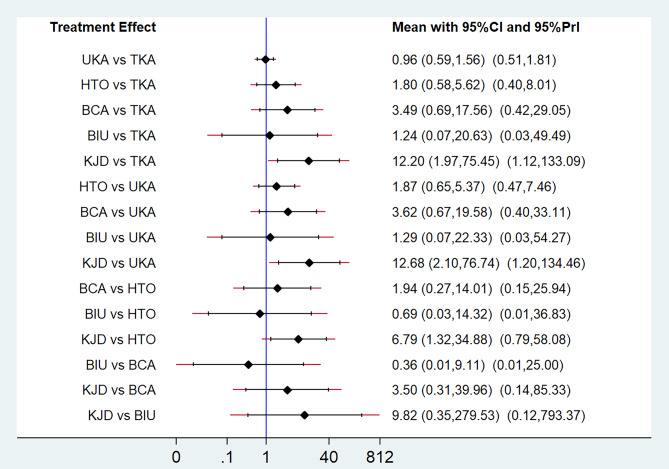
NMA results for reoperations. The treatments are compared in a forest plot. The vertical reference line centered at 1 indicates statistical equivalence

**Fig. 6:**
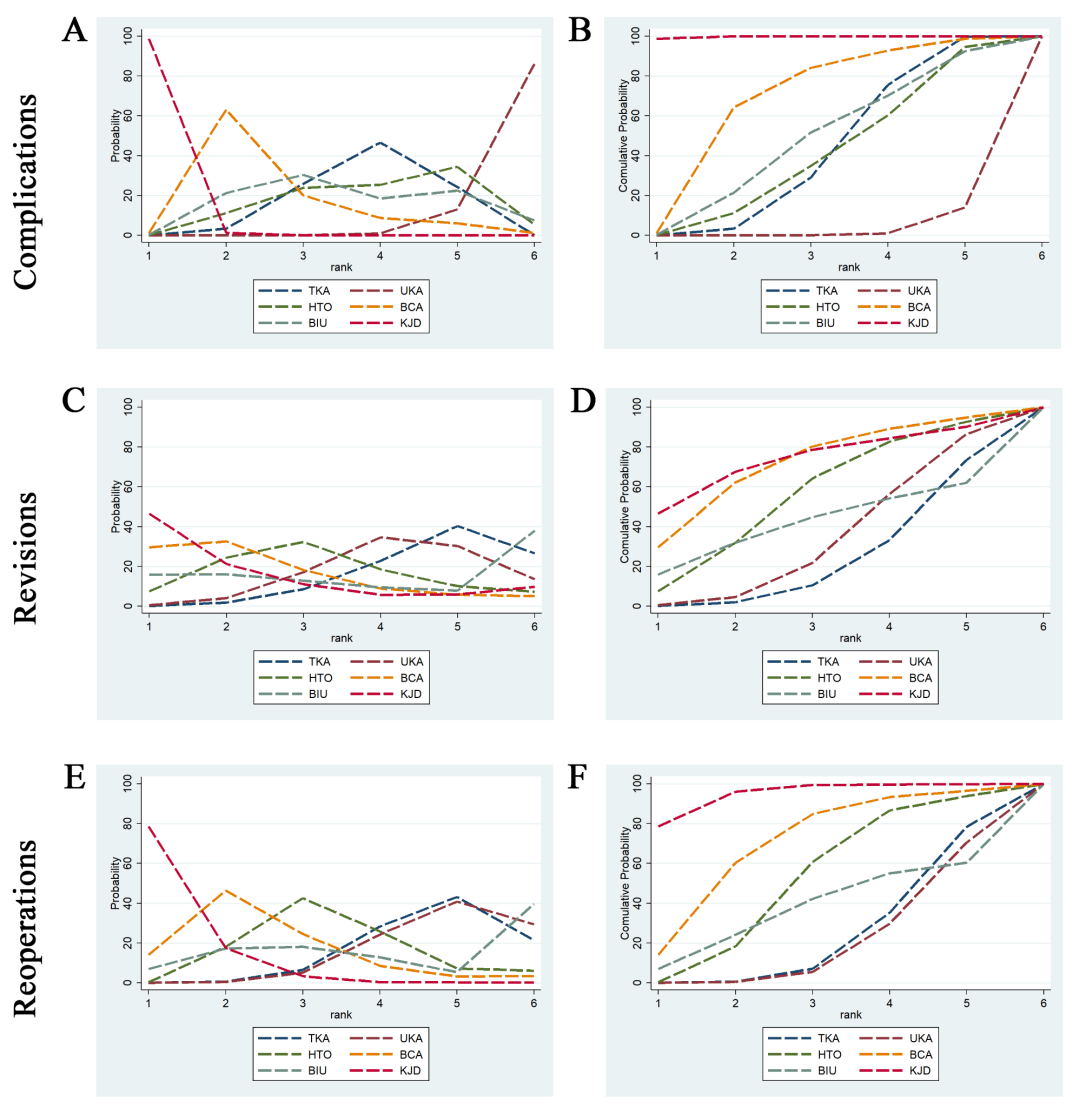
Ranking of different surgical interventions based on rank probabilities (**a, c, e**) and cumulative probabilities (**b, d, f**)

**Fig. 7:**
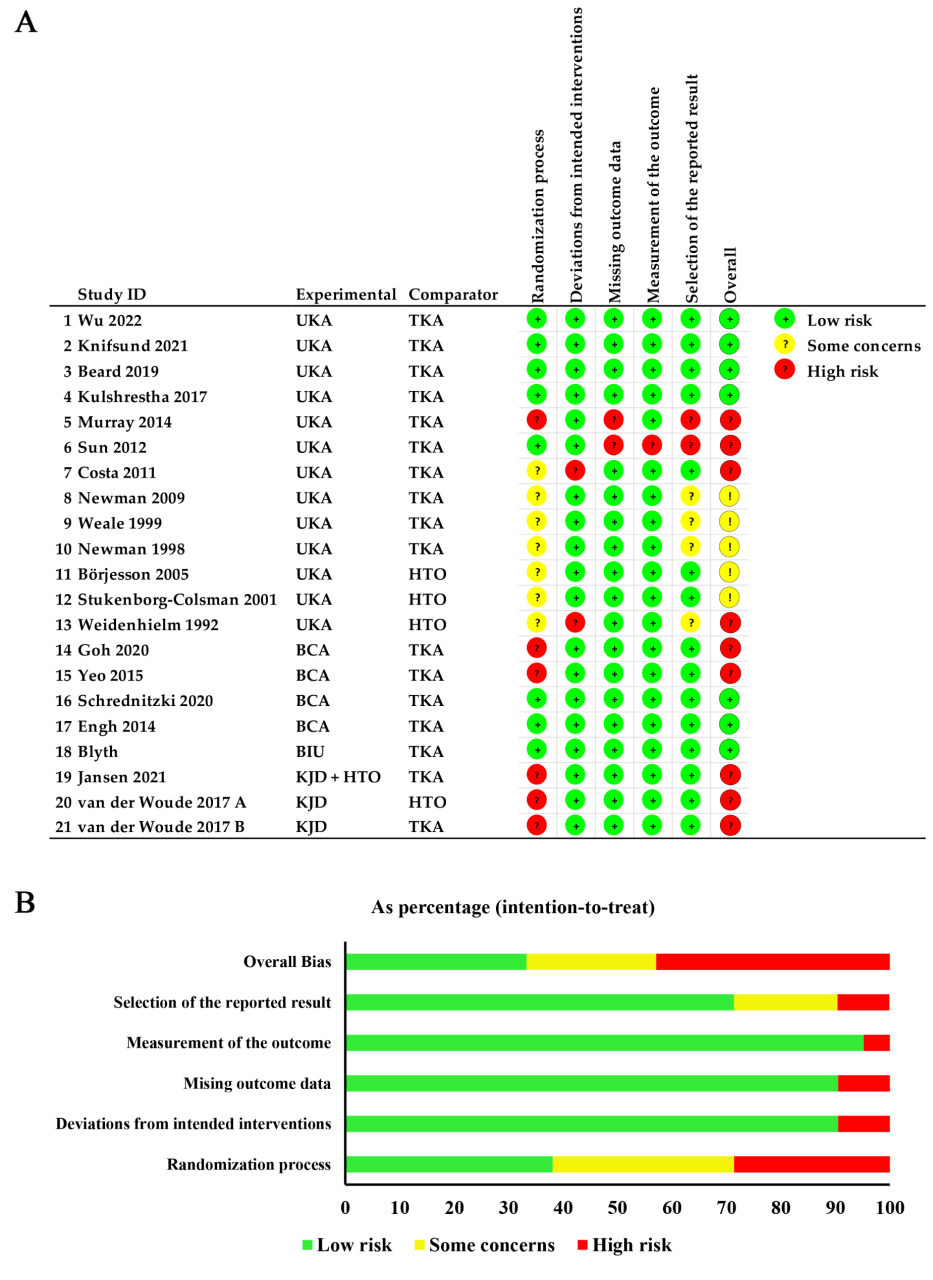
Risk of bias of the included studies (ROB2 bias assessment). a: each domain of studies with high, low, or unclear risk of bias and concerns regarding applicability; b: the proportions of studies with high, low, or unclear risk of bias and concerns regarding applicability

A network diagram summarizing the NMA geometry involving complications, revisions and reoperations is presented in Fig. 2A–C. In 14 studies [20–23, 25, 26, 29, 31–33, 35–38], the authors reported complications, and, in 13 studies [20–23, 25–27, 31, 33, 35–38], the authors reported revisions and reoperations. The results of the NMA, including the OR with 95% CIs, are reported in Figs. 3, 4 and 5, and the rank probabilities and cumulative probabilities are plotted in Fig. 6. Because an NMA involving functional outcomes was not possible due to the heterogeneity or deficiency of the functional data, a systematic review was performed for functional outcomes in all studies. The risk of bias of the RCTs is depicted in Fig. 7. Five studies (23.8%) showed some concerns, and 9 studies (42.9%) had a high risk for overall bias. In those RCTs, the majority of risk of biases arose from the randomization process, where it was unclear if the allocation sequence was random and concealed. All NMAs showed that heterogeneity and inconsistency were low.

### Complications

Complications were reported in 14 studies [20–23, 25, 26, 29, 31–33, 35–38]. The NMA revealed that the likelihood of complications for each method was as follows: KJD (99.8%), BCA (68.0%), BIU (47.7%), TKA (41.6%), HTO (40.0%), and UKA (3.0%) (Fig. 6A and B). These findings indicate KJD had the greatest probability of postoperative complications, followed by BCA, while UKA had the lowest probability of postoperative complications. There were statistically significant differences in the incidence of complications when performing the following comparisons: KJD vs. TKA, UKA, HTO, BCA, and BIU, UKA vs. TKA, and BCA vs. UKA (Fig. 3). The above NMA results are consistent with direct comparisons among different interventions (Fig. 3 and Supplementary Fig. 1).

### Revisions and reoperations

Revisions were reported in 13 studies [20–23, 25–27, 31, 33, 35–38]. The NMA SUCRA rankings indicating the likelihood of revisions were as follows: KJD (73.5%), BCA (71.3%), HTO (55.8%), BIU (41.7%), UKA (33.9%), and TKA (23.8%) (Fig. 6C and D), but there was no difference among all treatments (Fig. 4). No differences in the risk of revision among different treatments were observed with direct-comparison meta-analysis and NMA (Fig. 4 and Supplementary Fig. 2).

Reoperations were also reported in 13 studies [20–23, 25–27, 31, 33, 35–38]. The NMA SUCRA rankings indicating the likelihood of reoperations were as follows: KJD (94.8%), BCA (69.9%), HTO (52.0%), BIU (37.8%), TKA (24.3%), and UKA (21.2%) (Fig. 6E and F). There were statistically significant differences in the incidence of reoperations when comparing KJD with TKA, UKA, and HTO (Fig. 5). The above NMA results are consistent with the direct comparison among the different interventions (Fig. 5 and Supplementary Fig. 3).

### Functional outcomes

Functional outcomes were reported in all studies. The follow-up durations of these RCTs ranged from was 1 to 15 years. The majority of comparisons among various treatments showed no difference for functional outcomes in Table 2. Compared with TKA, only one RCT (1/8) definitely showed that UKA had a better functional enhancement after 3 years of follow-up [20] and three RCTs (3/8) revealed that UKA had a superior ROM (range of motion) [20, 21, 29]. For UKA vs. HTO, the functional outcomes were not different in all 3 RCTs [30–32]. During a follow-up period of 1 to 10 years, BCA and BIU resulted in similar clinical and functional scores as TKA in 5 studies [33–37]. KJD was noninferior to TKA and HTO in the primary functional outcomes in 3 studies [38–40], but a high incidence of pin track infection associated with KJD was found.

## Discussion

To date, there has been no consensus regarding the best surgical option for symptomatic knee osteoarthritis. To assist in providing robust evidence to guide surgical selection for surgeons and patients, the current NMA of 21 studies involving 1749 participants compared 6 surgical interventions (TKA, UKA, HTO, BCA, BIU, and KJD) and revealed that UKA and TKA are better options to treat symptomatic knee osteoarthritis, according to SUCRA rankings. KJD has the highest incidence of complications, revisions, and reoperations, thereby limiting its application. To our knowledge, this is the first NMA to compare the impacts of six surgical methods on the treatment of symptomatic knee osteoarthritis.

Some similar findings were also observed in other assessments of syntheses studies:(1) Most evidence-based studies showed that UKA is a better option than TKA, such as Wilson et al. who reported that UKA had significantly shorter operating times and hospital stays, fewer complications, and quicker recovery, but the revision rates for TKA were low [41]; Migliorini et al. and Arirachakaran et al. also showed that UKA reported reduced survivorship but better clinical and functional performances than TKA [42, 43]; Chawla’s meta-analysis indicated that revisions of medial UKA and lateral UKA occur at an annual rate of 2.18 and 2.31-fold that of TKA, respectively [44]; Only one meta-analysis showed that there were no statistically significant differences between UKA and TKA in terms of function scores, complications and survivorships, but they still supported the routine use of UKA for OA because of shorter hospital stay, faster recovery and less need for rehabilitation [45]; and Tripathy et al. reported that patients with UKA are better able to forget about their artificial joint than patients with TKA [46]. The reason why the rate of revision of TKA is lower than that of UKA is controversial. Revision of unsatisfactory TKA has a lower frequency because the reason for unsatisfactory results is also not known, but replacing with another TKA is risky when the reason for revisions is unknown and the solution is the same as the original. Most unsatisfactory UKA can be revised with TKA, so the relatively high revision rate of UKA can also be explained. In addition, UKA also has an obvious drawback in which the revised UKA to TKA had inferior outcomes compared to those of the primary TKA; hence, primary TKA may be a preferable procedure to UKA for patients for whom UKA and TKA are both applicable [47–49]. (2) Several meta-analyses demonstrated that UKA resulted in better clinical outcomes, greater improvement in physical activity levels, and fewer postoperative complications than HTO [50–52], and HTO is more appropriate for younger patients whereas UKA is appropriate for older patients [52, 53]; The Postoperative rate of revision and complications did not differ significantly between UKA and HTO [54]. Nevertheless, TKA following HTO provides similar clinical outcomes compared to TKR without previous HTO, but the conversion process of HTO to TKR is technically challenging [13, 55, 56]. (3) The majority of meta-analyses showed that BCA did not prove to be an equivalent alternative to TKA in knee OA due to more postoperative complications and poorer long-term survivorship [15, 57, 58]. (4) Wada’s and Takahashi’s systematic reviews reported that bi-UKA or KJD is a feasible and viable surgical option for knee OA in carefully selected patients, but long-term outcomes remain limited [17]. Moreover, 74% of KJD patients had complications, and 56% experienced pin tract infections [38].

Combined with the aforementioned literature review and the analysis of this NMA, most surgical methods for patients with OA have little difference in postoperative functional recovery of the knee joint; hence, patients and surgeons should pay more attention to complications, revisions, and reoperations when multiple options are available. According to our analysis, UKA is the preferred treatment for medial compartment OA patients with appropriate indications, followed by TKA. HTO is suitable for younger patients. Because there is little evidence for the applications of BCA, BIU and KJD, their indications need to be strictly controlled. Clinical decision-making for surgical options of patients with OA is meant to be informed by the best medical evidence, clinical judgment, surgeon’s experience and patient requirements. Treatment options should be carefully considered for each patient in accordance with their individual requirements and actual condition.

### Limitations

Our study has some limitations. First, to improve the quality of this NMA, only RCTs were included, which resulted in a small number of studies. There were few RCTs included, which might reduce the robustness of our conclusions, especially for BIU and KJD evaluations. Second, it is difficult to conduct an ideal RCT as a result of clinical ethics. The overall allocation process was unclear in most studies, leading to a moderate to high risk of selection bias. Third, these included RCTs varied in inclusion criteria, population demographics, end assessment points and methods, and varying degrees of statistical adjustment. Fourth, implant survival and revision rates are likely to be affected by the experience of the operating surgeon and hospital performing the procedure. Fifth, because only BIU was involved in lateral compartment OA, stratified analysis was not performed according to the number of affected compartments. Sixth, the conclusion is limited by the selection of reviewed publications and individual variation of surgical indications for patients. The strengths of this NMA are the comprehensive nature of the literature search along with the strict eligibility criteria, and only high-quality RCTs were included in the analysis.

## Conclusions

This study may provide evidence to support informed shared decision-making in the care of patients with knee OA. Based on our analysis, any surgical intervention is noninferior to other treatments in functional outcomes, but UKA and TKA are better options to treat symptomatic knee osteoarthritis according to SUCRA rankings by comparing complications, revisions, and reoperations. KJD is an imperfect option for treating OA. It needs to be emphasized that other treatment options should be carefully considered for each patient in accordance with their individual requirements and actual condition. Moreover, further well-designed and large-scale clinical trials and systemic reviews are required to confirm our findings.

## Electronic supplementary material

Below is the link to the electronic supplementary material.

Supplementary Material 1

Supplementary Material 2

Supplementary Material 3

Supplementary Material 4

Supplementary Material 5

## Data Availability

The datasets used and analyzed during the current study are available from the corresponding author on reasonable request.

## Abbreviations

RCT: randomized clinical trials
TKA: total knee arthroplasty
UKA: unicompartmental knee arthroplasty
HTO: high tibial osteotomy
BCA: bicompartmental knee arthroplasty
BIU: bi-unicompartmental knee arthroplasty
KJD: knee joint distraction
NMA: network meta-analysis
OA: osteoarthritis
PRISMA: Preferred Reporting Items for Systematic Reviews and Meta-Analyses
ROB2: the revised Cochrane risk of bias tool for randomized trials
Cis: confidence intervals
SUCRA: the surface under the cumulative ranking curve
ROM: range of motion
