# Supplementary material for: Surgical interventions for symptomatic knee osteoarthritis: a network meta-analysis of randomized control trials
Source: BMC Musculoskelet Disord. 2023 Apr 22;24:313. doi: 10.1186/s12891-023-06403-z (PMC10122318; doi:10.1186/s12891-023-06403-z)
Supplement: Supplementary file 3 — Supplementary Material 3 [file 12891_2023_6403_MOESM3_ESM.pdf]

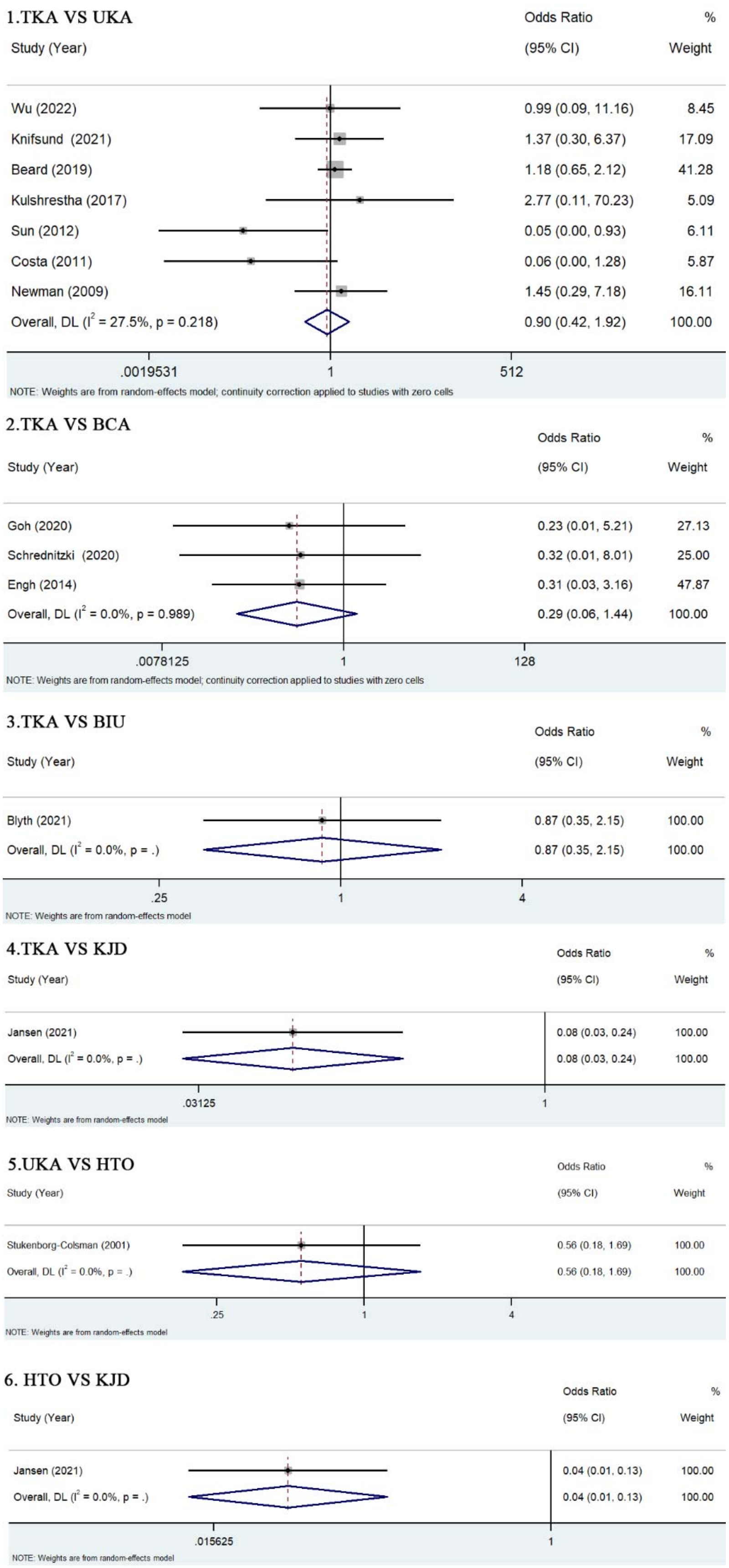

Supplementary Fig. 3. A forest plot of the traditional pairwise meta-analysis for reoperations between different surgical interventions.
