# Supplementary material for: Surgical interventions for symptomatic knee osteoarthritis: a network meta-analysis of randomized control trials
Source: BMC Musculoskelet Disord. 2023 Apr 22;24:313. doi: 10.1186/s12891-023-06403-z (PMC10122318; doi:10.1186/s12891-023-06403-z)
Supplement: Supplementary file 4 — Supplementary Material 4 [file 12891_2023_6403_MOESM4_ESM.pdf]

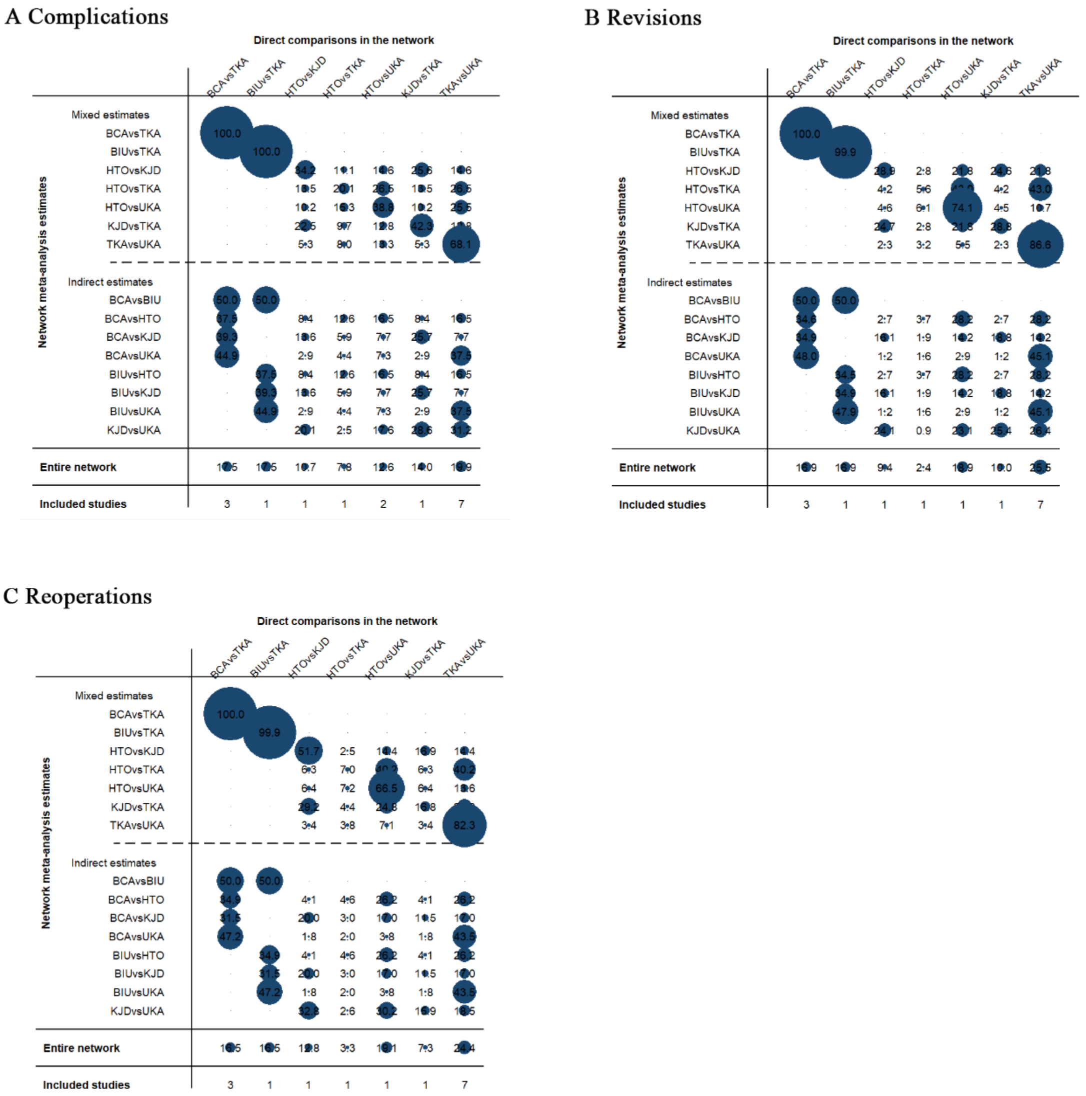

Supplementary Fig. 4. Contribution plot for network. The size of each circle is proportional to the weight attached to each direct summary effect for the estimation of each network summary effects.
